# Access, barriers, and quality of life: Student perspectives on disability support in higher education

**DOI:** 10.1371/journal.pone.0360516

**Published:** 2026-10-15

**Authors:** Hatem A. Alkhamra, Sana M. N. Abu Dahab, Rana A. Alkhamra

**Affiliations:** 1 Department of Counseling and Special Education, School of Educational Sciences, The University of Jordan, Amman, Jordan; 2 Department of Occupational Therapy, School of Rehabilitation Sciences, The University of Jordan, Amman, Jordan; 3 Department of Occupational Therapy, College of Rehabilitation Sciences, University of Manitoba, Manitoba, Canada; 4 Department of Speech and Language Pathology, School of Rehabilitation Sciences, The University of Jordan, Amman, Jordan; Aga Khan University Hospital, PAKISTAN

## Abstract

**Purpose:**

The purpose of this study was to examine students’ perspectives on access to disability support services, perceived barriers to service utilization, and quality of life within public higher education contexts. By examining these dimensions within the same study design and participant sample, the study aimed to provide a broader student-centered understanding of disability support and well-being in higher education.

**Design/methodology/approach:**

A cross-sectional survey design was employed with 79 students with disabilities who were formally registered with disability or student support services at two public universities in the Middle East, located in Qatar and Jordan. Data were collected using a questionnaire assessing perceived access to accommodations and barriers to service utilization, alongside the World Health Organization Quality of Life–BREF (WHOQOL-BREF). Nonparametric statistical analyses were conducted to examine differences in student-reported experiences across the two institutional contexts.

**Results:**

Students reported moderate overall access to disability-related accommodations and support services (Mdn = 1.95, IQR = 1.81–2.17) and moderate perceived barriers to service utilization (Mdn = 1.65, IQR = 1.47–2.00). Significant between-context differences were observed in access to selected accommodations, perceived barriers, and quality-of-life domains. Notable differences included quiet examination rooms (*U* = 249.00, *p* < .001, *r* = .65), physical health quality of life (*U* = 245.00, *p* < .001, *r* = .59), environmental quality of life (*U* = 251.00, *p* < .001, *r* = .58), and financial barriers to accessing services (*U* = 326.00, *p* < .001, *r* = .55). Overall, the findings indicate that students’ experiences of disability support varied across contexts in ways that were reflected in both service access and quality-of-life outcomes.

**Conclusion:**

This study contributes original evidence by examining access to disability support services, barriers to utilization, and quality of life within the same study design and participant sample. By foregrounding students’ perspectives and providing contextually grounded evidence from underrepresented higher education settings, the study offers valuable baseline insights to inform inclusive policy and service development in higher education.

## Introduction

Access to higher education for students with disabilities (SWD) has increased globally over recent decades, reflecting broader commitments to equity, inclusion, and participation [1,2]. Despite this progress, enrollment alone does not guarantee meaningful inclusion. Students with disabilities continue to encounter structural, attitudinal, and procedural barriers that shape their academic experiences, access to support services, and overall well-being [3–5]. Understanding these experiences from the students’ own perspectives is therefore essential for informing inclusive practices in higher education.

Internationally, inclusive higher education is guided by rights-based frameworks, most notably the United Nations Convention on the Rights of Persons with Disabilities (CRPD), which affirms the right of persons with disabilities to access higher education on an equal basis with others [6]. While the CRPD provides a global reference point, its implementation varies substantially across national and institutional contexts. As a result, universities differ in how disability support services are structured, resourced, and delivered, leading to variability in students’ access to accommodations and support [7,8].

Within higher education, disability support typically includes academic accommodations, assistive technologies, physical accessibility measures, and advisory or counseling services [9]. However, research consistently shows that the availability of services does not necessarily translate into effective utilization. Students with disabilities often report barriers related to limited awareness of services, complex administrative procedures, inadequate faculty support, negative attitudes, and concerns about stigma or disclosure [10–13]. Such barriers may restrict students’ participation and contribute to unequal educational experiences, even in institutions that formally endorse inclusive policies.

Beyond access to services, the broader university experience of students with disabilities is closely linked to quality of life. Quality of life encompasses physical, psychological, social, and environmental dimensions, all of which may be influenced by students’ interactions with institutional support systems [14]. Previous studies have demonstrated that insufficient accommodations, persistent barriers, and unsupportive environments can negatively affect students’ well-being, sense of belonging, and academic persistence [15–17]. Despite this, quality of life remains underexplored in higher education disability research, particularly in relation to students’ access to and experiences with institutional support services.

Research on disability support in higher education has expanded in recent years; however, much of the existing literature remains concentrated in Western contexts [18,19]. There is a relative scarcity of empirical studies examining student-reported experiences of disability support in higher education systems within the Middle East. Existing studies from the region suggest variability in service provision and highlight ongoing challenges related to accessibility, awareness, and institutional support [20,21]. Moreover, few studies have examined how institutional context—particularly differences in structural organization and resource availability—shapes students’ perceptions of access, barriers, and quality of life.

To address this gap, the present study examines student perspectives on disability support services, perceived barriers, and quality of life within two public universities operating in distinct higher education contexts in the Middle East. This study does not seek to compare institutional quality or rank universities. Rather, it aims to explore how students with disabilities experience access to support and navigate barriers within different structural and resource environments. By foregrounding student-reported experiences, the study seeks to contribute contextually grounded evidence that can inform inclusive policy development and service provision in higher education [15].

Barriers to accessing disability support services in higher education are not only administrative or procedural in nature; they have broader implications for student engagement and academic persistence. Research indicates that when accommodations are delayed, inconsistently applied, or insufficiently supported by faculty and administrative systems, students with disabilities may experience increased academic stress, reduced participation, and diminished sense of belonging within the university environment [5,15]. Over time, these experiences may influence students’ motivation, retention, and overall satisfaction with higher education [2]. Examining barriers alongside access to services is therefore essential for understanding how institutional practices shape students’ academic trajectories and well-being.

### Context of the study

The present study was conducted in two public universities located in distinct higher education contexts in Qatar and Jordan. Both institutions operate within national legal frameworks that formally recognize the rights of persons with disabilities to access higher education, consistent with the principles articulated in the United Nations Convention on the Rights of Persons with Disabilities [6]. In both contexts, higher education institutions are mandated to provide reasonable accommodations and facilitate access to educational services for students with disabilities in accordance with national legislation [22,23].

Despite shared legal commitments to disability inclusion, the two institutional contexts differ in terms of historical development, scale, and resource environments, which may influence the organization and delivery of disability support services. One institution is a long-established public university operating within a comparatively resource-constrained higher education system and serving a large and diverse student population. The other is a public university situated within a high-income national context, characterized by substantial investment in higher education infrastructure and ongoing system-level reform initiatives. These contextual differences may shape both the availability of support services and students’ experiences of accessing accommodations.

These differing structural and resource environments provide an opportunity to examine how participants reported access to support services, perceived barriers, and quality of life within varied higher education settings. Importantly, this study does not seek to compare institutional quality, evaluate performance, or rank universities. Rather, the inclusion of two distinct contexts allows for an exploration of student-reported experiences of disability support within different institutional conditions, consistent with prior research emphasizing the influence of institutional context on access, service provision, and student outcomes in higher education [7,8].

### Aim of the study

The aim of this study is to examine students’ perspectives on disability support in higher education, with a particular focus on perceived access to accommodations and support services, barriers to service utilization, and quality of life. By drawing on student-reported data from two public universities operating within distinct structural and resource contexts, the study seeks to explore how institutional environments shape the experiences and well-being of students with disabilities. This focus aligns with prior research emphasizing the importance of understanding disability support through students’ perspectives rather than solely through institutional policy or service descriptions [3,15].

### Research questions

1. How do students with disabilities perceive their access to disability-related accommodations and support services in higher education?
2. What barriers do students with disabilities report in accessing and utilizing accommodations and support services?
3. How do students with disabilities report their quality of life across physical, psychological, social, and environmental domains within the higher education context?
4. To what extent do perceptions of access, barriers, and quality of life vary across the two institutional contexts examined in this study?

## Methodology

### Participants

The study population consisted of students with disabilities who were formally registered with disability or student support services at two public universities in the Middle East, one in Qatar and one in Jordan. In both institutions, eligibility for disability-related support services requires the submission of official medical or educational documentation verifying the presence of a disability, in accordance with national regulations and institutional policies [22,23]. Only students who had completed this documentation process were eligible to participate in the study.

The inclusion of the two participating institutional contexts in Jordan and Qatar was intended to examine student-reported disability support experiences across settings that share regional and legal commitments to disability inclusion but differ in resource environments, institutional structures, and service-delivery conditions. This comparison was not designed to evaluate institutional quality or rank universities; rather, it was used to explore how differing contextual conditions may be reflected in students’ reported access to accommodations, perceived barriers, and quality-of-life outcomes.

Based on institutional records, it was initially anticipated that approximately 200 students with disabilities were receiving support services at each university. However, participation was voluntary, and recruitment was influenced by the sensitivity of disability disclosure in higher education contexts. As a result, a total of 79 students consented to participate in the study, including 37 students from Qatar and 42 students from Jordan.

Participants represented a range of disability types, including visual impairments, physical impairments, learning disabilities, and mental or psychological conditions, as documented by university support services. Disabilities were classified based on institutional records and self-reported confirmation by participants. Demographic characteristics of the sample are presented at the beginning of the Results section.

### Instruments

Two instruments were used to collect data for the present study: (a) the World Health Organization Quality of Life–BREF (WHOQOL-BREF) and (b) a researcher-developed questionnaire assessing access to disability support services and perceived barriers to service utilization.

#### Quality of life measure.

Quality of life was assessed using the World Health Organization Quality of Life–BREF (WHOQOL-BREF), a widely used self-report instrument developed by the World Health Organization to measure individuals’ perceptions of their position in life within their cultural and environmental context [14]. The WHOQOL-BREF consists of 26 items covering four domains: physical health, psychological well-being, social relationships, and environment. Responses are rated on a 5-point Likert scale, with higher scores indicating better perceived quality of life.

The WHOQOL-BREF has demonstrated robust psychometric properties across diverse cultural contexts, including Arabic-speaking populations. Arabic versions of the instrument have shown acceptable to strong reliability and validity in multiple studies conducted in the Middle East, supporting its use for assessing quality of life among university students and adult populations in the region [24–27]. In the present study, the WHOQOL-BREF was selected due to its multidimensional structure, cross-cultural applicability, and relevance for examining well-being in relation to environmental and institutional factors affecting students with disabilities.

#### Disability support services and barriers questionnaire.

The researcher-developed questionnaire was designed to assess students’ perceptions of access to disability-related accommodations and perceived barriers to service utilization in higher education. Its development was informed by existing literature on disability support services, accessibility, accommodation practices, and barriers experienced by students with disabilities in postsecondary education [28–32]. Items were selected and adapted to reflect common forms of academic, technological, physical, administrative, and attitudinal support or barriers reported in prior studies, while also allowing contextual relevance to the higher education settings examined in Jordan and Qatar.

The questionnaire consisted of three sections. The first section collected demographic information, including gender, academic level, type and onset of disability, prior receipt of support services, and academic performance indicators. The second section assessed students’ use of disability-related accommodations and support services (21 items), while the third section focused on perceived barriers to accessing and utilizing these services (17 items). Items addressed a range of academic, physical, technological, and administrative supports commonly reported in the literature on disability support in higher education.

Responses to the accommodations and barriers items were recorded using a 3-point Likert-type scale (1 = Low, 2 = Moderate, 3 = High). The use of a 3-point scale was intended to reduce response burden and facilitate clarity in self-reporting, particularly for respondents with visual impairments or other disabilities that may affect survey completion, a consideration noted in prior disability research employing simplified response formats [28,32].

To establish content validity and contextual appropriateness, the questionnaire was reviewed by a panel of ten experts specializing in special education, educational psychology, rehabilitation, and educational measurement from universities in Jordan and Qatar. Panel members evaluated the clarity, relevance, cultural appropriateness, and comprehensiveness of the items, as well as the adequacy of the demographic section. Based on expert feedback, minor revisions were made, including wording modifications, the addition of one item to the accommodations section, and the removal of three items from the barriers section. No separate pilot study was conducted; however, the expert review process was used to strengthen content validity, clarity, and contextual fit prior to administration. No additional psychometric validation, such as factor analysis, measurement invariance testing, or separate validation across the two institutional groups or disability categories, was conducted. Accordingly, the questionnaire should be interpreted as a contextually reviewed researcher-developed measure rather than as a fully validated cross-context instrument.

Internal consistency reliability was assessed using Cronbach’s alpha. The accommodations section yielded a reliability coefficient of 0.81, while the barriers section demonstrated a coefficient of 0.78, indicating acceptable internal consistency for both scales [33].

### Procedure

#### Ethical considerations.

Ethical approval for this study was obtained from the relevant institutional review boards in Qatar and Jordan prior to data collection. The study was conducted in accordance with the principles of the Declaration of Helsinki. Participation was voluntary, and written informed consent was obtained from all participants prior to data collection. Participants were enrolled university students who were formally registered with disability or student support services and were able to review the study information and provide informed written consent independently.

Capacity to consent was determined through participants’ status as enrolled university students, their ability to understand the study information presented to them, and their voluntary completion of the informed consent process. The consent procedure, including obtaining written informed consent directly from participants, was reviewed and approved by the relevant institutional review boards. No separate formal capacity assessment was conducted because the study involved anonymous survey research with enrolled university students, and disability status alone was not considered an indication of impaired capacity to consent. Confidentiality and anonymity were ensured throughout the research process, and all data were collected anonymously and analyzed in de-identified form.

Following IRB approval, coordination was established with disability and student support service units at each institution to facilitate participant recruitment. These units assisted in identifying eligible students and disseminating information about the study.

Participants were invited to take part in the study through individual and small-group meetings organized with the assistance of trained undergraduate research assistants. During these meetings, students were provided with a brief overview of the study objectives and procedures and were informed that participation was entirely voluntary.

Informed consent was obtained from all participants prior to data collection. Participants were assured of confidentiality and anonymity, and no identifying information was collected as part of the survey process. Questionnaires were administered in accessible formats to accommodate participants’ needs, including large-print materials and assistance with reading items when requested. Completed questionnaires were collected and stored securely, and all data were handled in accordance with institutional ethical guidelines.

To further reduce the risk of deductive re-identification in this relatively small sample, potentially identifying demographic characteristics were not included in the publicly shared dataset, and results are reported in aggregate form.

### Data analysis

Data were analyzed using the Statistical Package for the Social Sciences (SPSS), version 25. Descriptive statistics, including frequencies and percentages, were calculated to summarize participants’ demographic characteristics and responses to survey items.

Given the ordinal nature of the data derived from the 3-point Likert-type scale used in the accommodations and barriers questionnaire, as well as the relatively small sample size, nonparametric statistical tests were deemed appropriate for group comparisons. Prior to conducting inferential analyses, data distribution was examined using Shapiro–Wilk tests in addition to inspection of skewness, kurtosis, and distribution patterns. Several variables demonstrated departures from normality. Accordingly, the Mann–Whitney U test was used to examine differences in students’ perceptions of access to disability-related accommodations, perceived barriers, and quality of life domains across the two institutional contexts. Nonparametric tests such as the Mann–Whitney U are recommended when data do not meet assumptions of normality and when response categories are ordinal rather than continuous [34,35].

For the WHOQOL-BREF, domain scores were calculated according to World Health Organization scoring procedures [14]. The negatively phrased items (Q3, Q4, and Q26) were reverse scored before calculation of domain scores. Item scores were then averaged within each domain, multiplied by four, and transformed to a 0–100 scale using the WHO transformation formula, with higher scores indicating better perceived quality of life. The physical health domain comprised Q3, Q4, Q10, Q15, Q16, Q17, and Q18; the psychological domain comprised Q5, Q6, Q7, Q11, Q19, and Q26; the social relationships domain comprised Q20, Q21, and Q22; and the environment domain comprised Q8, Q9, Q12, Q13, Q14, Q23, Q24, and Q25. Q15 was missing for two participants; their physical health scores were calculated from the remaining six valid domain items. All 79 participants consequently had valid domain scores and were retained in the analyses; no cases were excluded because of missing WHOQOL-BREF data.

Results of the Mann–Whitney U analyses are reported using mean rank values, U statistics, standardized Z values, two-tailed p-values, and effect size *r*. Mean ranks were retained because they represent the standard comparative output for Mann–Whitney U tests and indicate the relative ordering of responses between groups rather than absolute mean scores. Effect size *r* was calculated as the absolute value of Z divided by the square root of the total sample size. Effect sizes were interpreted using conventional benchmarks, with *r* values of approximately  .10 considered small,  .30 moderate, and  .50 large [36]. Statistical significance was evaluated at α = 0.05, and all statistical tests were two-tailed. Where appropriate, descriptive summaries are reported using frequencies, percentages, medians, and interquartile ranges to support interpretation of the nonparametric findings. For descriptive interpretation of the accommodations and barriers scores, values were interpreted in relation to the original 3-point response scale (1 = Low, 2 = Moderate, 3 = High). References to low, moderate, or high levels therefore reflect proximity to the scale response categories rather than externally validated cut-off values.

## Results

The results are presented in four main sections. First, demographic characteristics of the sample are summarized. Second, overall patterns of access, barriers, and quality of life are reported. Third, findings related to students’ perceived access to disability-related accommodations and support services are presented. Fourth, perceived barriers to accessing and utilizing support services and differences in quality-of-life domains across the two institutional contexts are presented.

Table 1 summarizes the demographic characteristics of the sample by country. The final sample included 79 students with disabilities, with 37 participants from Qatar and 42 from Jordan. Across both contexts, visual impairment represented the largest disability category, followed by physical impairments, learning disabilities, and mental or psychological conditions. Participants were distributed across different academic levels and colleges, and most had received support services before entering higher education. These characteristics provide important context for interpreting the findings, particularly given the predominance of students with visual impairments in the sample.

**Table 1:** Demographic characteristics of participants by country.

		Qatar		Jordan	
		Frequency	Percentage	Frequency	Percentage
College	College of Arts	20	54.1	16	38.1
	College of Business	4	10.8	–	–
	College of Education	2	5.4	26	61.9
	College of Engineering	2	5.4	–	–
	College of Islamic Studies	9	24.3	–	–
Gender	Male	10	27.0	16	38.1
	Female	27	73.0	26	61.9
Disability	Learning Disabilities	2	5.4	–	–
	Physical impairments	6	16.2	14	33.3
	Visual Impairment	26	70.3	28	66.7
	Mental / Psychological Problems	3	8.1	–	–
Onset Of Problem	Before School	19	51.4	34	81.0
	In School	13	35.1	6	14.3
	After School	5	13.5	2	4.8
Year	Freshman	10	27.0	14	33.3
	Sophomore	10	27.0	2	4.8
	Junior	10	27.0	10	23.8
	Senior	3	8.1	12	28.6
	Late Graduate	4	10.8	4	9.5
Receiving Services Before College	Yes	32	86.5	28	66.7
	No	5	13.5	14	33.3
GPA	0 – 1	5	13.5	2	4.8
	1.1–2	7	18.9	14	33.3
	2.1 – 3	20	54.1	16	38.1
	3.1 – 4	5	13.5	10	23.8

### Overall patterns of access, barriers, and quality of life

Across the full sample, students reported moderate overall access to disability-related accommodations and support services, with an overall median access score of 1.95 (IQR = 1.81–2.17) on the 3-point scale. Perceived barriers were also present at a moderate level, with an overall median barrier score of 1.65 (IQR = 1.47–2.00). Quality-of-life domain scores, reported on the 0–100 WHOQOL-BREF scale, showed variability across domains. The highest median score was observed for social relationships (Mdn = 75.00, IQR = 50.00–93.75), followed by psychological well-being (Mdn = 62.50, IQR = 41.67–75.00), environment (Mdn = 59.38, IQR = 46.88–75.00), and physical health (Mdn = 50.00, IQR = 35.71–60.71). These overall findings provide descriptive context for the comparisons between the two participating institutional groups.

### Perceived access to disability support services

Students’ perceptions of access to disability-related accommodations and support services varied across the two institutional contexts. Overall, students in both settings reported moderate levels of access to a range of academic, technological, and physical accommodations, with statistically significant differences observed for several accommodation types.

Statistically significant differences were most evident for examination-related accommodations and mobility-related supports. Students at the participating institution in Qatar reported higher access to extended examination time (U = 343.00, p < .001, r = .54), quiet examination rooms (U = 249.00, p < .001, r = .65), reader/writer or word-processing support during examinations (U = 474.00, p = .001, r = .37), additional break time (U = 540.00, p = .013, r = .28), small-group support (U = 503.00, p = .004, r = .32), and mobility or transportation assistance on campus (U = 383.00, p < .001, r = .46). In contrast, students at the participating institution in Jordan reported significantly higher access to selected reading and technology-related supports, including recorded reading support (U = 562.00, p = .012, r = .28), electronic readers (U = 481.00, p = .001, r = .37), and dictionary use (U = 556.00, p = .015, r = .27). Effect sizes ranged from small to large, with the strongest differences observed for quiet examination rooms, extended examination time, and mobility or transportation assistance.

Table 2 presents descriptive and inferential statistics for perceived access to disability support services across the two institutional contexts.

**Table 2:** Descriptive and inferential statistics for perceived access to disability support services across institutional contexts.

Item	Qatar Mdn (IQR)	Jordan Mdn (IQR)	Qatar mean rank	Jordan mean rank	U	Z	r	p-value
Class presentations adjusted for students’ needs	3.00 (2.00–3.00)	2.00 (1.00–3.00)	44.78	35.79	600.00	−1.869	0.21	.062
Reading aloud by an assistant person	2.00 (2.00–3.00)	2.00 (1.00–2.00)	44.41	36.12	614.00	−1.705	0.19	.088
Read aloud by computer	1.00 (1.00–2.00)	2.00 (1.00–3.00)	36.14	43.40	634.00	−1.590	0.18	.112
Read aloud by recorder	1.00 (1.00–1.00)	1.00 (1.00–3.00)	34.19	45.12	562.00	−2.519	0.28	.012*
Read aloud by electronic reader	1.00 (1.00–1.00)	2.00 (1.00–3.00)	32.00	47.05	481.00	−3.308	0.37	.001*
Directions read aloud	1.00 (1.00–2.00)	2.00 (1.00–2.00)	37.65	42.07	690.00	−0.952	0.11	.341
Dictionary use	1.00 (1.00–3.00)	2.00 (1.00–3.00)	34.03	45.26	556.00	−2.426	0.27	.015*
Large print of materials	2.00 (1.00–3.00)	2.00 (1.00–2.00)	41.72	37.60	676.00	−0.861	0.10	.389
Scheduling appointments according to student schedule	3.00 (2.00–3.00)	2.00 (1.00–3.00)	45.92	34.79	558.00	−2.343	0.26	.019*
Extended time for the exam	3.00 ( 3.00–3.00)	2.00 (1.00–3.00)	51.73	29.67	343.00	−4.787	0.54	< .001*
Quiet non-distracting rooms for exams	3.00 (3.00–3.00)	2.00 (2.00–2.00)	54.27	27.43	249.00	−5.795	0.65	< .001*
Reader/writer assistant or word processor for exams	3.00 (3.00–3.00)	2.00 (1.00–3.00)	48.19	32.79	474.00	−3.262	0.37	.001*
Replace paper-and-pencil examinations with oral exams	1.00 (1.00–3.00)	1.00 (1.00–2.00)	39.30	40.62	751.00	−0.288	0.03	.773
Spelling and grammar aids for exams	1.00 (1.00–2.00)	2.00 (1.00–2.00)	35.32	44.12	604.00	−1.949	0.22	.051
Calculator use for exams	1.00 (1.00–3.00)	2.00 (1.00–2.00)	38.46	41.36	720.00	−0.611	0.07	.541
Additional break times	2.00 (1.00–3.00)	2.00 (1.00–2.00)	46.41	34.36	540.00	−2.479	0.28	.013*
Physical accommodations	2.00 (2.00–3.00)	2.00 (1.00–3.00)	44.68	35.88	604.00	−1.827	0.21	.068
Small-group support	2.00 (2.00–3.00)	1.00 (1.00–2.00)	47.41	33.48	503.00	−2.871	0.32	.004*
Assistive technology in writing or recording	2.00 (1.00–3.00)	2.00 (1.00–3.00)	40.89	39.21	744.00	−0.345	0.04	.730
Writing services	2.00 (1.00–3.00)	2.00 (1.00–2.00)	44.16	36.33	623.00	−1.622	0.18	.105
Mobility and transportation assistance on campus	3.00 (2.00–3.00)	1.00 (1.00–2.00)	50.65	30.62	383.00	−4.117	0.46	< .001*

**Note.** Qatar: n = 37; Jordan: n = 42; Mdn = median; IQR = interquartile range; r = effect size calculated as |Z|/√N. *p < .05.

### Perceived barriers to accessing support services

Students’ perceptions of barriers to accessing and utilizing disability support services are summarized in Table 3. Across both institutional contexts, students reported experiencing multiple barriers, though the pattern and intensity of reported barriers varied.

**Table 3:** Descriptive and inferential statistics for perceived barriers to disability support services across institutional contexts.

Item	Qatar Mdn (IQR)	Jordan Mdn (IQR)	Qatar mean rank	Jordan mean rank	U	Z	r	p-value
I do not use services because of personal reasons	1.00 (1.00–2.00)	2.00 (1.00–3.00)	31.89	47.14	477.00	−3.186	0.36	.001*
I do not use services because of shyness from others	1.00 (1.00–2.00)	2.00 (1.00–2.00)	34.81	44.57	585.00	−2.112	0.24	.035*
I am unable to defend and advocate my personal rights as a person with disability	1.00 (1.00–1.00)	2.00 (1.00–2.00)	32.08	46.98	484.00	−3.434	0.39	.001*
Lack of awareness by individuals	1.00 (1.00–2.00)	2.00 (2.00–3.00)	29.78	49.00	399.00	−3.943	0.44	< .001*
Lack of instructor assistance in providing accommodations	1.00 (1.00–2.00)	2.00 (2.00–2.00)	28.43	50.19	349.00	−4.703	0.53	< .001*
Lack of knowledge of how and where to obtain such services	1.00 (1.00–2.00)	2.00 (1.00–2.00)	35.30	44.14	603.00	−1.856	0.21	.063
Failure of adaptations to prove their effectiveness	2.00 (1.00–2.00)	2.00 (1.00–2.00)	34.84	44.55	586.00	−2.032	0.23	.042*
Lack of knowledge of laws or services	1.00 (1.00–1.00)	2.00 (1.00–2.00)	32.54	46.57	501.00	−2.988	0.34	.003*
Assistance requires considerable time, effort, paperwork, and correspondence	2.00 (1.00–3.00)	2.00 (2.00–2.00)	35.59	43.88	614.00	−1.729	0.19	.084
Problems at the university level in providing appropriate accommodations	1.00 (1.00–2.00)	2.00 (2.00–3.00)	28.92	49.76	367.00	−4.376	0.49	< .001*
Difficulty obtaining necessary papers to prove need/disability	1.00 (1.00–1.00)	2.00 (1.00–2.00)	29.81	48.98	400.00	−4.156	0.47	< .001*
Financial difficulties preventing access to paid services	1.00 (1.00–1.00)	2.00 (2.00–2.00)	27.81	50.74	326.00	−4.903	0.55	< .001*
Availability of accommodations/services	1.00 (1.00–1.00)	2.00 (2.00–3.00)	27.86	50.69	328.00	−4.764	0.54	< .001*
Challenges associated with student mobility and orientation	1.00 (1.00–2.00)	2.00 (2.00–2.00)	34.05	45.24	557.00	−2.309	0.26	.021*
Accessibility of the support system in relation to location, time, and schedule	1.00 (1.00–2.00)	2.00 (1.00–2.00)	34.95	44.45	590.00	−2.027	0.23	.043*
Attitudes toward persons with accommodations and willingness to provide accommodations	2.00 (1.00–2.00)	2.00 (1.00–2.00)	40.32	39.71	765.00	−0.129	0.01	.897
Decision makers’ awareness of the importance of providing accommodations	2.00 (1.00–2.00)	2.00 (2.00–2.00)	36.73	42.88	656.00	−1.315	0.15	.189

**Note.** Qatar: n = 37; Jordan: n = 42; Mdn = median; IQR = interquartile range; r = effect size calculated as |Z|/√N. *p < .05.

Students at the participating institution in Jordan reported significantly higher perceived barriers across several structural, administrative, and resource-related items. The largest differences were observed for financial difficulties preventing access to paid services (U = 326.00, p < .001, r = .55), limited availability of accommodations or services (U = 328.00, p < .001, r = .54), lack of instructor assistance in providing accommodations (U = 349.00, p < .001, r = .53), problems at the university level in providing appropriate accommodations (U = 367.00, p < .001, r = .49), and difficulty obtaining required documentation to prove the need for accommodations (U = 400.00, p < .001, r = .47). Students at the participating institution in Jordan also reported higher barriers related to lack of awareness by individuals (U = 399.00, p < .001, r = .44), difficulty advocating for personal rights as a person with a disability (U = 484.00, p = .001, r = .39), lack of knowledge of laws or services (U = 501.00, p = .003, r = .34), mobility and orientation challenges (U = 557.00, p = .021, r = .26), and accessibility of the support system in relation to location, timing, and scheduling (U = 590.00, p = .043, r = .23). Effect sizes ranged from small to moderate-to-large, indicating larger between-group differences in several structural and procedural barriers, with higher reported barrier levels at the participating institution in Jordan.

These results suggest that while barriers to service utilization exist in both contexts, the nature of these barriers differs, with structural and resource-related challenges more pronounced in one context and attitudinal concerns more salient in the other.

### Quality of life

Students’ perceived quality of life across physical health, psychological well-being, social relationships, and environmental domains is presented in Table 4. Statistically significant differences were observed across all four domains. Students in the participating institution in Qatar reported significantly higher quality-of-life scores across all four WHOQOL-BREF domains than students in the participating institution in Jordan. The largest difference was observed in physical health (*U* = 245.00, *p* < .001, *r* = .59), followed by environment (*U* = 251.00, *p* < .001, *r* = .58), psychological well-being (*U* = 269.00, *p* < .001, *r* = .56), and social relationships (*U* = 472.00, *p* = .002, *r* = .34). These effect sizes ranged from moderate to large, with the strongest between-group differences observed for physical health, environment, and psychological well-being.

**Table 4:** Descriptive and inferential statistics for quality-of-life domains across institutional contexts.

Domain	Qatar Mdn (IQR)	Jordan Mdn (IQR)	Qatar mean rank	Jordan mean rank	U	Z	r	p-value
Physical health	60.71 (53.57–78.57)	39.29 (32.14–50.00)	54.38	27.33	245.00	−5.252	.59	< .001*
Psychological well-being	66.67 (62.50–79.17)	45.83 (37.50–62.50)	53.73	27.90	269.00	−5.009	.56	< .001*
Social relationships	87.50 (62.50–100.00)	62.50 (37.50–75.00)	48.24	32.74	472.00	−3.042	.34	.002*
Environment	71.88 (62.50–84.38)	50.00 (34.38–59.38)	54.22	27.48	251.00	−5.185	.58	< .001*

**Note.** Qatar: n = 37; Jordan: n = 42; Mdn = median; IQR = interquartile range; r = effect size calculated as |Z|/√N. *p < .05.

### Summary of findings

Taken together, the results demonstrate that students’ experiences of disability support in higher education vary across institutional contexts in terms of access to accommodations, perceived barriers, and quality of life. While both groups reported utilizing a range of support services, differences were evident in the types of accommodations most accessible, the barriers most frequently encountered, and overall well-being across multiple domains. These findings provide an empirical basis for describing differences in students’ reported experiences across the two participating institutional contexts, which is further explored in the Discussion section.

## Discussion

This study examined students’ perspectives on access to disability support services, perceived barriers to service utilization, and quality of life within two public higher education contexts. Overall, students reported moderate levels of access to a range of academic, technological, and physical accommodations, though patterns of access varied by accommodation type across the two institutional contexts. Differences were also observed in the nature and extent of perceived barriers, as well as across quality-of-life domains. Taken together, these findings underscore the importance of examining disability support in higher education through students’ reported experiences, rather than relying solely on institutional policy or service availability indicators [3,15]. Particular attention is given to findings with moderate-to-large effect sizes, as these differences may have greater practical relevance for understanding students’ access, barriers, and well-being. The discussion is organized around three interrelated areas: access to accommodations and support services, barriers to service utilization, and quality of life.

Because the analyses were cross-sectional and unadjusted, observed between-group differences are interpreted descriptively and should not be taken as evidence that institutional or contextual characteristics caused the observed outcomes.

Findings related to access to disability support services suggest that, while formal accommodations are available in both institutional contexts, students’ experiences of access are not uniform across service types. Moderate overall access indicates that disability support infrastructures are in place; however, variability across accommodations highlights differences in how specific services are delivered, prioritized, or communicated to students. Similar patterns have been reported in prior research, which demonstrates that the presence of disability services does not necessarily ensure consistent or equitable access across accommodation categories [9,12].

Differences in access to examination-related accommodations, in-class supports, and technology-based services were observed across service types that may be related to institutional procedures and service-delivery arrangements; however, the present design does not permit determination of the factors responsible for these differences. Examination accommodations, for example, often rely on centralized processes and standardized procedures, whereas technology-based supports may depend on availability, training, and individual initiative. Previous studies have shown that students’ perceptions of access are influenced not only by formal eligibility but also by the clarity of procedures, responsiveness of service units, and coordination between disability services and academic departments [28,32].

Importantly, these findings should be interpreted as reflections of student-reported access rather than evaluations of institutional quality. Students’ perceptions provide critical insight into how disability support services are experienced in practice, revealing potential gaps between policy intent and day-to-day implementation. Understanding such experiential differences is essential for informing inclusive service planning and ensuring that accommodations are accessible, timely, and responsive to students’ needs across diverse higher education contexts [7].

Findings related to perceived barriers indicate that, despite the availability of disability support services, students continue to encounter challenges that may limit effective utilization.

Across both institutional contexts, students reported barriers related not only to the availability of services but also to administrative procedures, communication processes, and the broader institutional climate. This reinforces existing evidence that barriers to disability support in higher education are often multifaceted and extend beyond the mere presence of formal accommodations [5,11].

The higher prevalence of structural and administrative barriers in one participating institution indicates that these barriers were reported more frequently in that institutional dataset; the present analyses do not establish why these differences occurred. Previous research has shown that cumbersome procedures and unclear pathways for accessing accommodations can discourage students from seeking support, particularly when processes are perceived as time-consuming or burdensome. Such barriers may disproportionately affect students who require timely adjustments to participate fully in academic activities [10,12,13].

In contrast, attitudinal and interpersonal barriers emerged as more salient in the other context, particularly those related to faculty responsiveness, awareness of disability-related needs, and concerns surrounding disclosure. Consistent with prior studies, students may hesitate to request accommodations when they anticipate negative reactions, lack of understanding, or inconsistent application of approved accommodations by instructors [10,32]. These findings underscore the importance of institutional climate and faculty engagement in facilitating—or constraining—students’ access to support.

Taken together, the pattern of reported barriers indicates that both structural and interpersonal factors were represented in students’ reports of access to disability support services. Even in contexts where services are formally available, barriers related to processes, attitudes, and communication can limit students’ ability to benefit fully from existing supports. Addressing these barriers requires attention not only to service provision but also to the institutional practices and interactions that influence how support is experienced by students with disabilities [7].

Access to disability support services and the barriers encountered in utilizing them have implications that extend beyond academic logistics to students’ broader experiences in higher education. When accommodations are delayed, inconsistently implemented, or difficult to access, students may experience increased stress, reduced participation, and a diminished sense of belonging within the university environment. Prior research suggests that such experiences can influence students’ engagement with coursework and campus life, as well as their overall satisfaction with higher education, even when formal supports are available [5,15]. Examining quality of life alongside access and barriers provides a more comprehensive description of students’ reported experiences across the participating institutional contexts.

Findings related to quality of life indicate statistically significant differences across physical, psychological, social, and environmental domains between the two institutional contexts, with the strongest differences observed in environmental, physical, and psychological quality of life. Quality of life is a multidimensional construct reflecting individuals’ perceptions of their well-being in relation to their health, social relationships, and surrounding environment [14]. Differences were observed across multiple quality-of-life domains; however, the present study cannot determine whether these differences are attributable to academic accommodations, environmental conditions, psychosocial factors, or other influences.

The environmental domain showed one of the strongest differences between the participating institutional groups. Although environmental quality of life encompasses factors such as physical accessibility, transportation, campus infrastructure, and supportive resources, the present analyses cannot determine which factors account for the observed difference. Previous studies have highlighted the sensitivity of environmental quality of life to contextual and institutional conditions, especially for individuals with disabilities who rely on accessible spaces and coordinated supports to participate fully in academic and campus life [9,16]. These findings underscore the importance of considering environmental conditions as an integral component of inclusive higher education.

Differences in psychological and social quality of life domains further suggest that students’ well-being is influenced by their sense of belonging, perceived support, and interactions within the academic community. Experiences related to stigma, disclosure, and faculty responsiveness—identified as barriers in the present study—may contribute to variations in these domains. Consistent with prior research, supportive institutional climates and positive interpersonal interactions have been associated with better psychological well-being and social integration among students with disabilities [4,10].

Importantly, quality of life outcomes should not be interpreted as direct consequences of disability support services alone. Students’ well-being is shaped by multiple intersecting factors, including personal circumstances, societal attitudes, and broader policy environments. Nevertheless, examining access, barriers, and quality of life within the same study highlights the value of incorporating well-being indicators into evaluations of disability support in higher education. Doing so allows for a more holistic assessment of inclusion that extends beyond service availability to encompass students’ overall experiences and participation within institutional contexts [15].

### Contribution of the study

First, this study contributes to the literature on disability support in higher education by examining three related but often separately studied dimensions within the same research design and participant sample: students’ perceived access to disability support services, perceived barriers to service utilization, and quality of life. Examining these dimensions concurrently provides a broader descriptive picture of students’ reported experiences across multiple aspects of disability support and well-being.

The findings should not be interpreted as evidence of causal relationships or statistical associations among access, barriers, and quality of life; rather, they describe patterns observed across these domains and variation between the participating institutional groups [3,15].

Second, the study provides contextually grounded evidence from two public higher education institutions operating within distinct structural and resource environments in the Middle East—a region that remains underrepresented in the higher education disability literature. By foregrounding student-reported experiences rather than institutional policies or service descriptions, the findings offer insight into how disability support is experienced in practice within different higher education contexts.

Third, the study advances understanding of disability support by illustrating that inclusion in higher education extends beyond the formal availability of accommodations. The findings draw attention to institutional processes, communication practices, and campus climate as relevant considerations when interpreting students’ reported access to services and quality of life. Together, these contributions underscore the importance of adopting a student-centered, context-sensitive approach when evaluating and developing disability support services in higher education.

### Practical implications and recommendations

The findings of this study have practical implications for higher education institutions seeking to strengthen disability support services and reduce barriers to participation. Given the cross-sectional and student-reported nature of the data, these recommendations should be interpreted as practice-oriented considerations rather than causal prescriptions.

Disability support units may benefit from reviewing and streamlining procedures related to documentation, accommodation requests, and service delivery. Simplifying administrative processes, improving communication about available supports, and ensuring timely implementation of accommodations may reduce barriers that discourage service utilization. Regular feedback mechanisms that capture students’ experiences can further support continuous improvement and responsiveness to students’ needs.

Faculty play a critical role in shaping students’ experiences of access and inclusion. Providing faculty with targeted training on disability awareness, accommodation processes, and inclusive teaching practices may help reduce attitudinal barriers and improve consistency in the implementation of approved accommodations. Clear communication between faculty and disability support services can also facilitate more effective coordination and support for students in academic settings.

At the institutional level, the findings highlight the importance of considering environmental and psychosocial factors as part of inclusive higher education planning. Investments in accessible infrastructure, transportation supports, and assistive technologies may contribute to improved environmental quality of life for students with disabilities.

Additionally, fostering inclusive campus climates that promote belonging and reduce stigma can support students’ psychological and social well-being. Institutions may consider incorporating quality of life indicators into evaluations of disability support services to capture the broader impact of inclusion initiatives beyond service uptake alone.

### Limitations

Several limitations should be considered when interpreting the findings of this study.

First, the sample was drawn from only two public universities, one in each country, and included students formally registered with disability or student support services. Accordingly, the findings apply to the participating institutional samples and should not be interpreted as representative of students with disabilities nationally in either Qatar or Jordan, or of students with disabilities who are not formally registered for support services.

Second, the study employed a cross-sectional design and relied on self-reported data, which precludes causal inferences and may be subject to response bias. Third, although students with different disability types may experience disability support services and institutional barriers differently, subgroup analyses were not conducted due to sample size limitations across disability categories. In addition, although the researcher-developed questionnaire demonstrated acceptable internal consistency and underwent expert review, its psychometric equivalence across institutional contexts and disability groups was not formally evaluated.

Relatedly, the sample was predominantly composed of students with visual impairments. As a result, the findings may disproportionately reflect the support needs and service experiences of students with visual impairments and may not fully represent those of students with other disability types, such as physical, hearing, intellectual, learning, or psychosocial disabilities. Different disability groups may encounter distinct institutional, environmental, and attitudinal barriers; therefore, the findings should be interpreted within the context of the disability distribution represented in the sample. Future research with larger and more diverse samples is needed to examine potential differences across disability groups.

Finally, the data for this study were collected during 2017–2018, prior to the COVID-19 pandemic and the widespread expansion of digital and hybrid learning environments in higher education. As a result, the findings should be interpreted as pre-pandemic baseline evidence rather than as a current assessment of disability support systems in Jordan and Qatar. Disability support services, accessibility technologies, and institutional practices may have changed substantially since data collection, particularly in relation to digital accessibility, remote learning accommodations, and online service delivery. Nevertheless, the findings remain valuable because they document structural, administrative, and attitudinal barriers that were present before these system-level changes and provide a basis for future research examining how disability support has evolved in the post-pandemic period.

## Conclusion

This study examined the perspectives of students formally registered with disability or student support services regarding access to accommodations, perceived barriers to service utilization, and quality of life within two participating public higher education institutions. The findings indicate that students reported differences across the two institutional groups in access to specific accommodations, perceived barriers, and quality-of-life domains. These findings are descriptive and should not be interpreted as evidence that institutional or contextual characteristics caused the observed differences. Rather, the study provides pre-pandemic baseline evidence from two participating institutional settings that can inform future research, inclusive service planning, and the continued examination of disability support in higher education. Future research involving larger and more diverse samples across multiple institutions is needed to determine the extent to which these patterns are observed in other higher education settings.

## Supporting information

S1 Checklist Checklist_Inclusivity-in-global-research-questionnaire. (DOCX)

S1 Data Data sheet. (XLSX)
